# Supplementary material for: Quantitative Real-Time RT-PCR Verifying Gene Expression Profile of Cavitations Within Human Jaw Bone
Source: Biomedicines. 2025 May 8;13(5):1144. doi: 10.3390/biomedicines13051144 (PMC12109557; doi:10.3390/biomedicines13051144)
Supplement: Supplementary file 1 [file biomedicines-13-01144-s001.zip › biomedicines-3535612-supplementary.pdf]

biomedicines

MDPI

Supplementary Materials:

Table S1. List of primers that were used for gene expression analyses including assay name and catalog number.

PRIMER ABREVATION	Gene	Assay name	Cat.no.
RANTES	Regulated And Normal T cell Expressed and Secreted	Hs_CCL5_1_SG QuantiTect Primer Assay	Oiagen QT000090083
BMP2	Bone morphogenic protein 2	Hs_BMP2_1_SG QuantiTect Primer Assay	Qiagen QT00012544
RANKL	Receptor Activator of NF-κB Ligand	Hs_TNFSF11_1_SG QuantiTect Primer Assy	Qiagen QT00215614
COL1	Collagen1	Hs_Col1A1_1_SG QuantiTect Primer Assay	Quiagen QT00037793
ICAM	Intercellular adhesion molecule 1	Hs_ICAM1_1_SG QuantiTect Primer Assay	Qiagen QT00074900
SELP	P-Selectin	Hs_SELP_1_SG QuantiTect Primer Assay	Qiagen QT00012516
BMP1	Bone morphogenic protein 1	Ha_BMP1_1_SG QuantiTect Primer Assay	QiagenQT00000819
COL10	Collagen10	Hs_Col10A1_1_SG QuantiTect Primer Assay	Qiagen QT00096348
COL14	Collagen14	Hs_Col14A1_1_SG QuantiTect Primer Assay	Qiagen QT00062426
COL18	Collagen18	Hs_Col18A1_1_SG QuantiTect Primer Assay	Qiagen QT01026445
SMA	smooth muscle actin	Hs_ACTA2_1_SG QuantiTect Primer Assay	Qiagen QT00088102
FLT1	Vascular endothelial growth factor receptor 1	Hs_FLT1_1_SG QuantiTect Primer Assay	Qiagen QT00073640
IGF1	Insulin-like growth factor	Hs_IGF1_1_SG QuantiTect Primer Assay	Qiagen QT00029785
LAMA	Laminin	Hs_LAMA1_1_SG QuantiTect Primer Assay	Qiagen QT00038318
SP7	transcription factor Sp7, Osterix	Hs_SP7_1_SG QuantiTect Primer Assay	Qiagen QT00213514
COL1	Collagen 1	Hs_Col1A1_2_SG QuantiTect Primer Assay	Qiagen QT02589482
COL2	Collagen 2	Hs_Col2A1_va_1_SG QuantiTect Primer Assay	Qiagen QT00998844
COL4	Collagen 4	Hs_Col4A1_1_SG QuantiTect Primer Assay	Qiagen QT00005250
ON	Osteonectin	Hs_Sparc_1_SG QuantiTect Primer Assay	Qiagen QT00018620
OC	Osteocalcin	Hs_BGLAP_!_SG QuantiTect Primer Assay	Qiagen QT00232771
SMA	smooth muscle actin	Hs_ACTA2_va.1_SG QuantiTect Primer Assay	QiagenQT02407307
KOR	kappa opioid receptor	Hs_OPRK1_1_SG QuantiTect Primer Assay	Qiagen QT00015316

 https://doi.org/10.3390/10.3390/biomedicines13051144
